# Exercise-secreted antitumor myokines: mechanistic insights and translational potential

**DOI:** 10.3389/fspor.2026.1830759

**Published:** 2026-05-28

**Authors:** Songyang Han, Cuiyao Du, Chenqiong Zhao, Qi Zhang, Meiling Wu, Cheng Yang, Xia Liu

**Affiliations:** 1 College of Sports Science and Health, Harbin Sport University, Harbin, China; 2 Sports Humanities and Sociology College, Harbin Sport University, Harbin, China

**Keywords:** cancer suppression, exercise, molecular mechanism, myokines, skeletal muscle

## Abstract

**Background:**

Cancer remains a major threat to human health. Exercise has been shown to reduce cancer risk, inhibit tumor progression, and improve patient prognosis and quality of life; however, its precise molecular underpinnings are not yet fully understood.

**Objective:**

To synthesize current evidence and identify critical knowledge gaps, this review focuses on exercise-induced myokines secreted by skeletal muscle and examines their potential direct and indirect roles in tumorigenesis and malignant progression.

**Findings:**

We systematically reviewed the molecular mechanisms by which several key exercise-responsive myokines exert tumor-suppressive effects, including interleukin-6 (IL-6), Secreted Protein Acidic and Rich in Cysteine (SPARC), irisin, and other prominent myokines.

**Results:**

Significant progress has been made in elucidating the antitumor mechanisms of major myokines. Nevertheless, their intracellular signaling pathways remain incompletely defined. The majority of existing studies rely on *in vitro* cell models and lack validation in physiologically relevant *in vivo* settings or clinical contexts. Notably, several myokines exhibit functional duality, capable of exerting either tumor-suppressive or tumor-promoting effects depending on the specific microenvironmental context.

**Conclusion:**

Future research must urgently delineate the interaction networks of myokines with their upstream regulators and downstream effectors. It is essential to validate their true *in vivo* mechanisms using standardized animal models and well-characterized clinical samples. Furthermore, systematic evaluation of their pharmacokinetics, delivery strategies, and potential off-target effects is required to advance the clinical translation of myokine-based therapeutic interventions.

## 1 Introduction

### 1.1 Research background on the current situation of cancer and the anticancer effect of exercise

Cancer represents a major global public health challenge and poses a serious threat to human health and life. In 2020, the International Agency for Research on Cancer (IARC) reported approximately 19.3 million new cancer cases and 10 million cancer-related deaths worldwide (1). In recent years, both cancer incidence and mortality have continued to rise, placing a substantial burden on societies and families. Symptoms such as pain, fatigue, and psychological distress, common during disease onset and treatment, significantly impair patients' quality of life. Therefore, cancer prevention and strategies to support patient recovery are of critical importance.

Evidence suggests that roughly 25% of global cancer cases are linked to modifiable lifestyle factors, including prolonged sedentary behavior, excess body weight, and physical inactivity (2). Regular physical activity has been consistently shown to reduce cancer risk, support recovery in patients, and lower cancer-related mortality (3, 4). Adults who engage in more than 150 min of moderate-intensity exercise or more than 75 min of vigorous-intensity exercise per week experience better overall health and a reduced risk of cancer (5). Even brief episodes of vigorous intermittent lifestyle physical activity (VILPA) demonstrate notable anticancer effects: a UK cohort study of 22,398 participants found that just 3–4 min of daily VILPA significantly lowered overall cancer risk, underscoring the potential importance of exercise intensity in mediating anticancer benefits (6). Long-term, regular physical activity has been associated with reduced incidence of multiple cancers. Numerous epidemiological studies report that higher levels of physical activity correlate significantly with lower risks of bladder, colon, breast, esophageal adenocarcinoma, endometrial, kidney, and stomach cancers, and that high activity levels are linked to a 40%–50% reduction in cancer-specific mortality (7–9).

A robust body of evidence confirms that exercise reduces cancer risk, enhances recovery, and decreases mortality. Diverse exercise intensities and modalities have demonstrated anticancer effects, providing a strong rationale for further investigation into the biological mechanisms underlying exercise-induced cancer protection. Current understanding holds that exercise inhibits tumor progression through multiple pathways, including modulation of body composition, alteration of obesity-related metabolic factors, and activation of the immune system (10). Among these mechanisms, exercise-responsive myokines, cytokines secreted by skeletal muscle, have emerged as a key area of research interest.

### 1.2 The concept and classification of myokines

Myokines are bioactive molecules produced and secreted by skeletal muscle into the bloodstream during contraction, particularly in response to exercise. They mediate intercellular communication and regulate physiological processes, including metabolism, immunity, and inflammation, through endocrine, paracrine, and autocrine mechanisms (11). Myokines can influence the tumor microenvironment (TME) directly or indirectly, thereby exerting anticancer effects (12). Based on structural and functional characteristics, muscle-derived factors are broadly categorized into three groups: growth factors (e.g., BDNF, IGF-1), cytokines (e.g., OSM, IL-6, IL-15), and other proteins [e.g., irisin, decorin, and Secreted Protein Acidic and Rich in Cysteine (SPARC)]. Each class of myokines contributes distinct anticancer functions in response to physical activity.

Investigating the molecular mechanisms by which exercise-responsive myokines counteract cancer will elucidate the intrinsic biological basis of exercise-induced antitumor effects and may yield novel conceptual frameworks and practical approaches for cancer prevention and therapy. This review summarizes key myokines released from exercise-responsive skeletal muscle and delineates their roles in modulating malignant phenotypes of tumor cells and influencing tumor progression. These insights offer a theoretical foundation for the development of myokine-based anticancer therapeutics and biologics, support the design of personalized exercise regimens for cancer patients in clinical settings, and hold considerable potential to enhance treatment efficacy and patient prognosis, thereby conferring significant clinical and societal value.

## 2 Materials and methods

To ensure a thorough, rigorous, and reproducible synthesis of the existing evidence, this study employs a systematic literature search combined with a standardized screening protocol. This approach is designed to identify high-quality studies directly relevant to the molecular mechanisms linking exercise, skeletal muscle–derived myokines, and tumor suppression.

### 2.1 Search strategy

A systematic literature search was conducted across three authoritative English-language electronic databases: PubMed, Web of Science, and Scopus. The search spanned from each database's inception through December 2025 to ensure comprehensive coverage and inclusion of the most recent findings. Boolean logic operators were used to combine key search terms as follows: (“exercise” OR “physical activity”) AND (“myokine” OR “muscle-derived factor”) AND (“cancer” OR “tumor” OR “neoplasm” OR “tumorigenesis”) AND (“IL-6” OR “OSM” OR “irisin” OR “decorin” OR “SPARC” OR “IL-15” OR “BDNF” OR “IGF-1”). In addition, the reference lists of relevant review articles and primary research studies were manually screened to identify eligible studies that may have been missed in the initial database search.

### 2.2 Research screening and inclusion and exclusion criteria

Search results were screened according to predefined criteria to ensure the relevance and methodological quality of the included studies.

Inclusion criteria encompassed peer-reviewed, full-text English-language articles that focused on exercise-regulated myokines and their antitumor effects. Eligible studies reported molecular mechanisms, *in vitro* experiments, animal experiments, or clinical evidence. Original research articles, systematic reviews, and mechanism-related reviews were included.

Exclusion criteria consisted of editorials, letters, opinion articles, case reports, conference abstracts, and preliminary research briefs. Studies lacking clear mechanistic or functional data, those not related to exercise, myokines, and cancer, as well as duplicate publications and low-quality research, were excluded.

To facilitate cross-study comparison and strengthen the methodological rigor of evidence synthesis, 20 representative eligible original studies were systematically summarized in Table 1. These studies were arranged chronologically by publication year and detailed the core information including author, year, study design, experimental model, and key findings regarding the antitumor mechanisms of exercise-responsive myokines.

**Table 1:** Key included studies on exercise-induced myokines and cancer.

Author/Year	Study design	Study model	Key findings
Santra et al., 1995 (13)	*In vitro* cellular assay; *in vivo* animal study	Human colon cancer WiDr cells; scid/scid mice	Ectopic expression of *decorin* suppresses the malignant phenotype of colon cancer cells and induces G1-phase cell cycle arrest, an effect that can be reversed by *decorin* antisense oligonucleotides.
Chicharro et al., 2001 (14)	Human exercise physiology study	17 professional cyclists during a 3-week endurance race; serum samples	Prolonged high-intensity endurance exercise increases serum levels of total IGF-I and IGFBP-1, while decreasing the concentration of free IGF-I.
Goldoni et al., 2008 (15)	*In vitro* cellular assay; *in vivo* animal study	ErbB2-overexpressing breast cancer cells; murine breast cancer xenograft model	*Decorin* induces apoptosis in ErbB2-positive breast cancer cells and exerts synergistic anti-tumor effects with AG879; systemic administration significantly inhibits tumor growth and metastasis.
Hojman et al., 2011 (16)	*In vitro* cellular assay; Exercise intervention	MCF-7/MDA-MB-231 breast cancer cells; 6-month training vs. acute exercise	Acute post-exercise serum significantly suppresses breast cancer cell viability, whereas long-term exercise training confers no obvious cumulative anti-tumor effect.
Aoi et al., 2012 (17)	*In vitro* cellular assay; *in vivo* animal study	Colon cancer cell lines; *SPARC*-knockout murine colon cancer model	*SPARC* overexpression inhibits the malignant phenotype of colon cancer cells, and regular exercise promotes apoptosis of colonic mucosal cells.
Zhang et al., 2012 (18)	*In vitro* cellular assay	Gastric cancer cell lines	*SPARC* overexpression downregulates the expression of VEGF and MMP-7, thereby inhibiting gastric cancer cell proliferation and tumor angiogenesis.
Kong et al., 2013 (19)	*In vitro* cellular assay	Human hepatocellular carcinoma SMMC-7721 cell line	Oncostatin M (OSM) inhibits hepatoma cell proliferation, induces apoptosis and G0/G1-phase cell cycle arrest, promotes cellular differentiation, and reduces AFP and γ-GT levels.
Shin et al., 2013 (20)	*In vitro* cellular assay; Tissue microarray	Prostate cancer cell lines; human prostate tissue microarray	Exogenous *SPARC* inhibits prostate cancer cell proliferation and migration by binding to integrin β1 and reduces phosphorylation of the AKT signaling pathway.
Pedersen et al., 2016 (21)	*In vivo* animal study	Multiple tumor models; female mice subjected to voluntary running	Voluntary running significantly reduces tumor incidence and growth rate, an effect dependent on epinephrine-mediated NK cell mobilization and IL-6-regulated NK cell redistribution.
Dethlefsen et al., 2016 (22)	*In vitro* cellular assay	MCF-7 breast cancer cell line	Exercise upregulates OSM levels in mouse serum and muscle tissue; OSM inhibits MCF-7 breast cancer cell proliferation and induces apoptosis.
Kurgan et al., 2017 (23)	*In vitro* cellular assay	Non-small cell lung cancer (NSCLC) cell lines	Serum following high-intensity interval training suppresses NSCLC cell proliferation by inhibiting the activation of the Akt/mTOR/p70S6 K/Erk1/2 signaling pathway.
Kong et al., 2017 (24)	*In vitro* cellular assay	A549 and NCI-H446 lung cancer cells	Irisin inhibits epithelial-mesenchymal transition (EMT) by regulating the PI3 K/AKT/Snail pathway, thereby attenuating lung cancer cell proliferation, migration, and invasion.
Liu et al., 2018 (25)	*In vitro* cellular assay	MIA PaCa-2 and Panc03.27 pancreatic cancer cells	Irisin inhibits pancreatic cancer cell proliferation in a dose-dependent manner, induces G1-phase arrest, and reverses EMT via the AMPK/mTOR signaling pathway.
Dudás et al., 2019 (66)	*In vitro* cellular assay	Head and neck squamous cell carcinoma (HNSCC) cell lines	BDNF induces EMT in HPV-positive HNSCC without enhancing chemoresistance, while improving tumor cell survival in HPV-negative HNSCC.
Huang et al., 2020 (26)	*In vitro* cellular assay; *in vivo* animal study; Clinical sample analysis	U87MG glioblastoma cells; murine GBM model; human GBM tissues	Irisin suppresses glioblastoma proliferation and invasion by inducing G2/M-phase cell cycle arrest and upregulating TFPI-2, and exhibits tumor-targeting properties.
Siersbæk et al., 2020 (27)	*In vitro* cellular assay; Nuclear transcriptional analysis	ER-positive breast cancer cells (MCF7, T47D, etc.)	The IL-6/STAT3 signaling pathway hijacks ER/FOXA1 enhancers to drive breast cancer metastasis, which can be effectively blocked by JAK inhibitors.
Kim et al., 2022 (28)	Human exercise intervention study	Patients with advanced metastatic castration-resistant prostate cancer (mCRPC) undergoing 6-month multimodal exercise; serum samples	Multimodal exercise elevates serum OSM and *SPARC* levels in patients with advanced prostate cancer, both of which significantly inhibit prostate cancer cell growth *in vitro*.
Nasiri et al., 2023 (29)	*In vivo* animal study	Murine pancreatic cancer model; *IL-18R*-knockout CD8+ T cells	The IL-18R signaling pathway inhibits the infiltration and migration of intratumoral CD8+ T cells; *IL-18R* knockout enhances T-cell infiltration efficiency and motility.
Stamatakis et al., 2023 (6)	Human exercise physiology study	22,398 adult participants; daily physical activity measured by accelerometer	Daily 3.4–3.6 min of vigorous non-exercise physical activity is independently associated with a 17%–18% significant reduction in overall cancer risk.
Nandakumar et al., 2025 (30)	*In vitro* cellular assay; Human tissue sample analysis	Prostate cancer cells; murine model; human primary prostate cancer tissues	IGF-1 drives immune escape in prostate cancer cells by suppressing antigen presentation and upregulating PD-L1 expression, and is closely associated with immunotherapy resistance.

## 3 The multifaceted role of skeletal muscle in antitumor defense

Skeletal muscle, the largest organ in the human body and the cornerstone of the motor system, not only generates mechanical force but also serves as a central regulator in diverse pathophysiological processes (31). Its antitumor effects are mediated through multiple mechanisms, including modulation of oxidative stress, remodeling of the metabolic microenvironment, and endocrine signaling.

Studies have demonstrated that exercise-induced skeletal muscle contractions generate high levels of oxidative stress within the muscle itself, establishing an intrinsic mechanism that suppresses tumor progression. Work from Cyrus M. Ghajar's team has shown that although metastatic tumor cells can migrate to skeletal muscle, they struggle to colonize and proliferate there. This resistance is attributed to the persistent oxidative stress imposed by the skeletal muscle microenvironment, which effectively curtails the proliferative potential of tumor cells (32). Furthermore, exercise stimulates skeletal muscle to secrete substantial amounts of lactate, markedly elevating local lactate concentrations. This may desensitize the vascular system to the additional lactate produced by invasive tumor cells, thereby impeding their proliferation within skeletal muscle (33). In addition, lactate has been shown to potentiate systemic antitumor immune responses and contribute to tumor immune surveillance (34).

Skeletal muscle also exerts a potent endocrine function. Exercise stimulates the release of multiple myokines that modulate metabolism and immune responses in distant tissues. *In vitro* studies have shown that, compared with serum collected at rest, human serum obtained after exercise markedly reduces the metabolic activity and proliferation of various tumor cell lines (35, 36), including those derived from prostate (28), breast (22), colon (37), pancreatic (38), and lung cancers (23). Animal experiments further demonstrated that breast cancer cells pretreated with post-exercise human serum exhibited substantially reduced tumorigenicity and led to significantly lower tumor incidence in mice (39). However, most of the aforementioned animal studies employed immunodeficient mouse models, which cannot fully recapitulate the human immune microenvironment and therefore inadequately reflect the regulatory effects of myokines in immunocompetent hosts. This limitation remains a major constraint in current *in vivo* research. These findings highlight the potential of muscle-derived secreted factors to influence antitumor immunity and shape the TME.

## 4 Regulation of myokine secretion by exercise

### 4.1 Exercise promotes the expression and secretion of muscle factors

Skeletal muscle–derived myokines act locally within the muscle and on neighboring tissues, and, via the circulation, exert effects on distant organs, thereby contributing to systemic physiological regulation (40–42). Exercise serves as the primary physiological stimulus for skeletal muscle, markedly enhancing myocellular metabolism and promoting fiber contraction, which in turn upregulates the expression and secretion of a wide array of muscle-derived factors (43, 44). Compared with the resting state, the synthesis and release of these factors increase substantially during exercise, giving rise to a dynamic endocrine response network.

Studies have demonstrated that exercise elevates the expression of several key myokines, including interleukin-6 (IL-6), interleukin-8 (IL-8), and interleukin-10 (IL-10). It also upregulates connective tissue growth factor (CTGF), leukemia inhibitory factor (LIF), brain-derived neurotrophic factor (BDNF), SPARC, oncostatin M (OSM), irisin, and decorin (45). These molecules collectively modulate inflammatory responses, energy metabolism, neural plasticity, and tumor cell behavior.

### 4.2 The role of extracellular vesicles in myokine release

Myokine release does not rely solely on the diffusion of free proteins; extracellular vesicles (EVs) may serve as an important transport pathway. EVs are classified by size into three subtypes: exosomes (30–150 nm), microvesicles (approximately 1 μm), and apoptotic bodies (1–5 μm). Although EVs were once considered mere byproducts of cellular metabolism, recent studies have established their role in mediating intercellular communication through autocrine, paracrine, or endocrine mechanisms (46).

**Figure 1:**
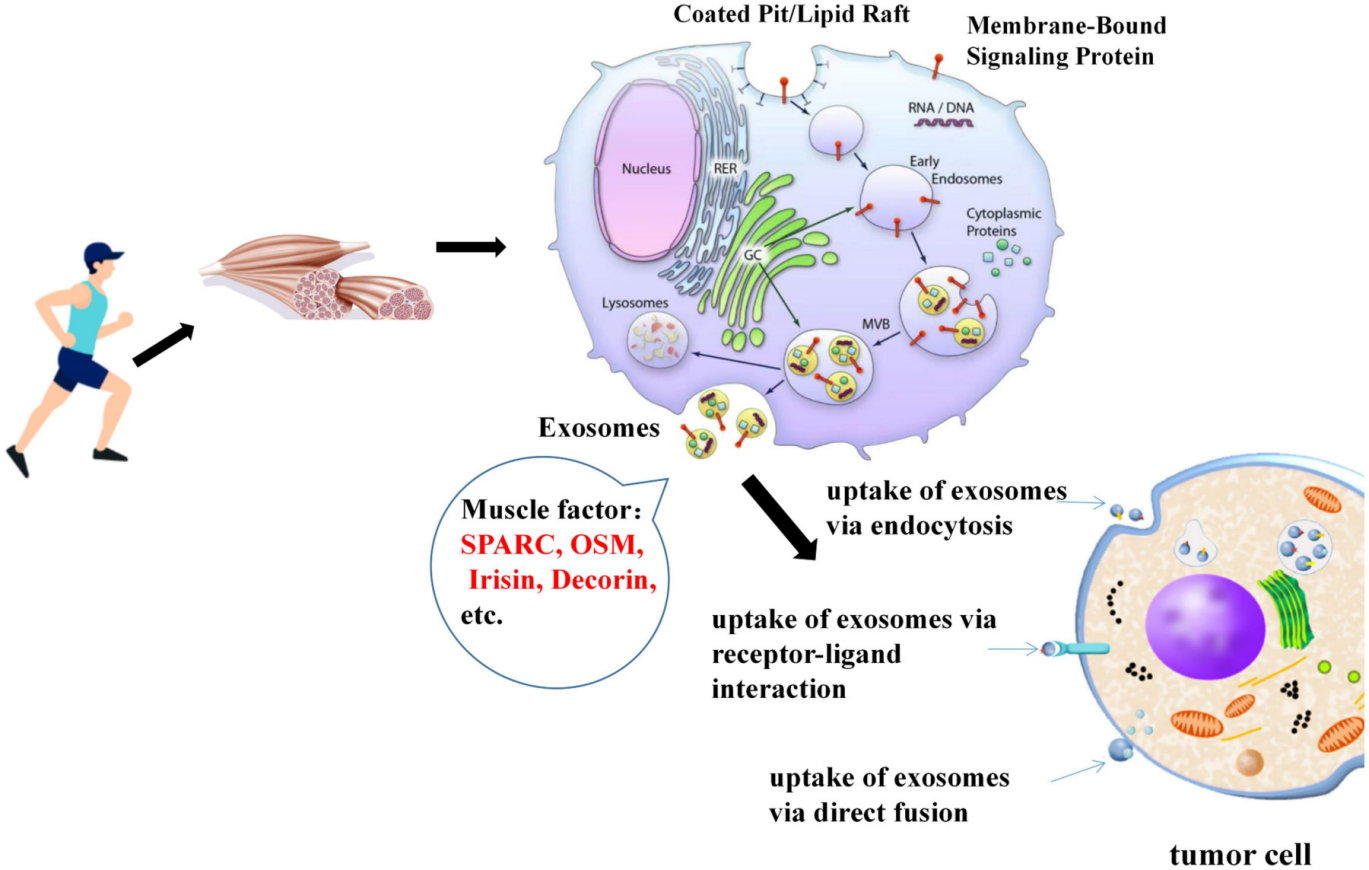
Exercise modulates exosome secretion from skeletal muscle to influence tumor development.

Exercise induces skeletal muscle cells to form early endosomes via inward budding of the plasma membrane. These endosomes then undergo intraluminal invagination to generate multivesicular bodies (MVBs), which subsequently fuse with the plasma membrane to release their intraluminal vesicles as EVs. To date, the loading and trafficking of exosomes have been extensively investigated in this context. Once released into the extracellular space, EVs carry myokines, peptides, chemokines, and hormones. Through endocytosis or binding to cell-specific receptors, they activate defined signaling pathways, thereby influencing neighboring or distant tissues and organs (47). The specific mechanism is illustrated in Figure 1. The influence of exercise on tumor cells is likely associated with EV-mediated delivery of exercise-responsive myokines. Upon entering tumor cells, these muscle-derived factors modulate signaling programs that regulate proliferation, invasion, and metastasis, thereby impeding tumor progression. Muscle-derived factors such as SPARC, OSM, irisin, and decorin may play key roles in this process.

## 5 Common motor response myokines and their tumor-suppressive molecular mechanisms

### 5.1 Cytokines: myokines

#### 5.1.1 Interleukin-6 (IL-6)

IL-6 was the first exercise-responsive myokine to be identified. During exercise, skeletal muscle contraction rapidly stimulates its secretion, with the amount released depending on exercise intensity, duration, and mode. In aerobic exercise, IL-6 levels increase markedly with higher intensity and longer duration and remain elevated for some time afterward; for example, serum IL-6 can rise several-fold following moderate-intensity aerobic exercise lasting more than 30 min (48). Resistance exercise also increases IL-6 secretion, although the pattern and magnitude differ from those observed with aerobic exercise: after an acute resistance session, IL-6 rises rapidly and then declines quickly. Low muscle glycogen levels before exercise further enhance exercise-induced IL-6 secretion.

Under specific conditions, IL-6 exhibits significant anti-cancer potential. *In vitro* evidence primarily supports three mechanisms. First, IL-6 activates the JAK/STAT3 signaling pathway, upregulates p21 and p27 expression, and induces G1 cell cycle arrest in cancer cells. The same pathway also downregulates oncogenes such as c-Myc and cyclin D1, thereby blocking proliferative signaling. Second, IL-6 triggers apoptosis via the mitochondrial pathway by promoting cytochrome c release and subsequent activation of caspase-9 and caspase-3. Additionally, IL-6 modulates immune cell function within the TME by enhancing the activation and proliferation of T cells and natural killer (NK) cells, which strengthens their cytotoxic activity.

However, other studies indicate that during tumor initiation and progression, IL-6 secreted by tumor cells and associated stromal cells drives oncogenic signaling through multiple synergistic pathways. By activating canonical pathways such as JAK/STAT3 and non-canonical pathways including MAPK, PI3 K/AKT, and NF-κB, IL-6 regulates tumor cell proliferation, survival, invasion, angiogenesis, and stemness maintenance (49). IL-6–induced STAT3 phosphorylation confers chemotherapy resistance by upregulating anti-apoptotic genes and mediating metabolic reprogramming (27, 50). In the TME, IL-6 suppresses dendritic cell maturation and differentiation, promotes macrophage polarization toward the M2 phenotype, and upregulates immune checkpoint molecules such as PD-L1, thereby facilitating immune evasion and diminishing responses to immunotherapy (51). Moreover, IL-6 exerts pathological effects even in epithelial-derived tumors lacking membrane-bound IL-6 receptor (IL-6R) by signaling through the soluble IL-6 receptor (sIL-6R). This trans-signaling mechanism activates stromal and immune cells via paracrine pathways, reinforcing a tumor-supportive inflammatory milieu (52).

The apparent contradiction in IL-6's function, its reported pro-tumor and anti-tumor effects, stems largely from limitations in current research approaches. This discrepancy arises primarily from systematic differences between simplified *in vitro* models and the complex physiology of *in vivo* systems. Chronic, sustained IL-6 exposure typically promotes tumor angiogenesis and epithelial–mesenchymal transition (EMT), contrasting sharply with the transient, pulsatile IL-6 release triggered by acute exercise, which exerts tumor-suppressive effects. Relying solely on *in vitro* data therefore oversimplifies IL-6's true pathophysiological role and fails to account for context-dependent outcomes shaped by cytokine source and secretion dynamics. It is thus critical to distinguish between IL-6 produced within the TME, a driver of chronic inflammation and tumor progression, and exercise-induced, skeletal muscle–derived IL-6, which acts as a key mediator of anti-tumor immunity. In contrast, IL-6 released from exercise-stimulated skeletal muscle enhances systemic anti-tumor immunity by mobilizing NK cells and boosting their cytotoxicity, thereby inhibiting tumor development (21).

#### 5.1.2 Oncostatin M (OSM)

OSM, a member of the IL-6 cytokine family, is secreted by activated T lymphocytes, monocytes, and macrophages. Its expression in skeletal muscle is substantial and significantly upregulated under exercise-induced conditions. Studies have shown that OSM inhibits tumor progression. Hojman et al. reported that treating MCF-7 breast cancer cells with serum from exercised mice markedly reduced cell proliferation (42% lower than control) and increased caspase activity (46% higher than control), indicating a proapoptotic effect. Neutralization with OSM-specific antibodies reduced caspase activity by 51%, further confirming OSM's role in mediating exercise-related antitumor effects (16).

OSM abnormally upregulates c-Myc transcription and protein expression by activating the STAT3 signaling pathway, which in turn activates the p53/p21 axis, induces G0/G1 arrest in the hepatoma cell lines SMMC-7721 and HepG2, and significantly suppresses their proliferative capacity (19). *In vitro* experiments have confirmed that OSM blocks tumor progression by both inhibiting proliferation and inducing apoptosis. However, *in vivo*, where skeletal muscle secretes large amounts of OSM following exercise, it remains unclear whether immune status, hypoxia levels, or other microenvironmental factors modulate OSM activity and preserve its anticancer effects. Therefore, *in vivo* studies are urgently needed to validate these findings.

#### 5.1.3 Interleukin-7 (IL-7) and interleukin-15 (IL-15)

IL-7 and IL-15 are key regulators of T cell homeostasis, and their secretion by skeletal muscle increases markedly after exercise (53). IL-7 inhibits apoptosis and promotes T cell survival through activation of the JAK/STAT5 and PI3K–AKT signaling pathways (54). It also enhances T cell receptor signal transduction, promotes peripheral T cell activation, and preserves the proliferative capacity of immature thymic T cells by downregulating the N-glycan GlcNAc branch on T cells, thereby maintaining immune function and homeostasis (55).

IL-15 exerts broad immunomodulatory effects by regulating the activation, proliferation, and effector functions of multiple immune cell types, including NK cells, memory CD8⁺ T cells, and NKT cells. Its antitumor activity is primarily mediated through the promotion of CD8⁺ T cell and NK cell activation and expansion (56, 57). Together, these cytokines constitute a critical component of the body's antitumor immune defense.

#### 5.1.4 Interleukin-1β (IL-1β) and interleukin-18 (IL-18)

Inflammatory mediators released by monocytes, such as IL-1β and IL-18, regulate transcriptional networks associated with malignant cell growth. Studies have shown that levels of both cytokines increase when tumor cells undergo pyroptosis. IL-1β and IL-18 are commonly used as biomarkers to assess antitumor effects. In colorectal cancer, their high expression in the TME is closely associated with suppression of malignant behavior and improved prognosis, thereby contributing to key antitumor outcomes (58, 59).

IL-18 is cleaved by caspase-3 to generate a 15 kDa short-chain isoform. Unlike the full-length protein, this variant does not bind IL-18Rα via the classical secretory pathway; instead, it translocates to the nucleus and upregulates ISG15 expression and secretion through CDK8-mediated phosphorylation of STAT1 at Ser727. This process activates highly cytotoxic NK cells, enabling them to exert antitumor effects. Notably, high nuclear expression of this short IL-18 isoform is strongly correlated with favorable prognosis in patients with colorectal cancer (60). IL-1β, the downstream core effector of the NLRP3 inflammasome, serves as a key regulator of antitumor immunity. Signaling through its receptor IL-1R, it contributes to the induction, differentiation, and functional regulation of cytotoxic T lymphocytes (CTLs). IL-1β cooperates with IL-18 in tumor immune regulation, and its expression level is closely linked to tumor suppression, thereby influencing disease progression (29).

Currently, systematic research is lacking on how exercise modulates skeletal muscle secretion of IL-1β and IL-18, including their secretion patterns, dependence on exercise intensity, temporal dynamics, and *in vivo* tumor-suppressive effects. The underlying mechanisms and clinical implications remain in an early exploratory phase. Future studies should focus on identifying the cellular sources, characterizing secretion profiles, and delineating the immune-regulatory networks of IL-1β and IL-18 during exercise interventions. Such work will address a critical gap in understanding the exercise–inflammation–muscle factor–antitumor axis.

### 5.2 Growth factor-like myokines

#### 5.2.1 Insulin-like growth factor-1 (IGF-1)

IGF-1 is a multifunctional growth factor secreted by skeletal muscle during exercise. It is generally believed to promote tumor cell proliferation and migration and to inhibit apoptosis through activation of the IGF-1R signaling pathway (30, 61). However, recent studies have increasingly identified contexts in which IGF-1 exerts tumor-suppressive effects. Notably, during exercise interventions, IGF-1 plays a significant role in regulating immune homeostasis and enhancing antitumor immune responses. Exercise influences IGF-1 secretion in a time- and intensity-dependent manner. Acute exercise induces a transient rise in serum IGF-1 that returns to baseline over time. For instance, following high-intensity acute exercise, IGF-1 levels increase immediately and then gradually decline within several hours. In contrast, long-term regular exercise leads to a sustained elevation in IGF-1; for example, 12 weeks of resistance training significantly increased serum IGF-1 levels (14).

A low-fat diet combined with exercise reduced serum IGF-1 and insulin levels while increasing IGFBP-1, thereby inhibiting proliferation and promoting apoptosis or necrosis of LNCaP prostate cancer cells *in vitro* (62). The role of IGF-1 in the TME is complex; nonetheless, existing evidence generally supports a predominant pro-tumorigenic function. Importantly, exercise, through precise modulation of timing and intensity, induces physiological fluctuations in IGF-1 that elicit immunoregulatory and antitumor effects distinct from those observed in pathological conditions (63). The specific mechanisms by which exercise modulates IGF-1's anticancer activity, the effective concentration thresholds, and interindividual variability remain unclear and warrant further investigation. Thus, IGF-1 represents a promising target for exercise-mediated cancer suppression, and deeper mechanistic insights are needed to strengthen the scientific foundation for exercise-based interventions.

#### 5.2.2 Brain-derived neurotrophic factor (BDNF)

BDNF is widely expressed in both the nervous system and skeletal muscle, and its secretion is markedly enhanced by exercise. Both aerobic and resistance exercise elevate serum BDNF levels, which correlate positively with exercise intensity and duration. Long-term, moderate-intensity aerobic exercise leads to a sustained increase in circulating BDNF (64). Within the TME, BDNF exhibits pronounced biological pleiotropy.

BDNF promotes tumor progression by activating the RAS/MAPK and PI3K/AKT pathways through its receptor, the tyrosine kinase TrkB. This signaling enhances the survival of colorectal cancer cells under stress and protects them from anoikis. In head and neck squamous cell carcinoma (HNSCC), paracrine BDNF signaling from cancer-associated fibroblasts (CAFs) drives EMT and confers chemotherapy resistance (65, 66). Furthermore, BDNF exerts proangiogenic effects primarily via hypoxia-inducible factor-1α (HIF-1α), particularly in hypoxic microenvironments.

Conversely, BDNF also demonstrates antitumor activity. The antisense long noncoding RNA BDNF-AS epigenetically suppresses tumorigenesis. In glioma, BDNF-AS interacts with DNA methyltransferase 1 (DNMT1), reducing methylation of the NEDD4L promoter and thereby promoting ubiquitination and degradation of YAP1. This cascade ultimately inhibits VEGFA expression and angiogenesis (67). Additionally, BDNF directly enhances the cytotoxic function of T and NK cells via TrkB, stimulating secretion of IFN-γ and perforin (66).

This dual role underscores the necessity of tailoring BDNF-targeted strategies to specific tumor contexts. For instance, in HPV-negative HNSCC, inhibition of the BDNF/TrkB axis reverses chemotherapy resistance, whereas in glioma, activation of the BDNF-AS/NEDD4L pathway shows therapeutic promise.

### 5.3 Other protein-based myokines

#### 5.3.1 Cysteine-rich acidic secreted protein (SPARC)

SPARC is one of the most extensively studied myokines associated with cancer. Studies show that a single bout of exercise can markedly increase SPARC release from skeletal muscle into the circulation in both healthy humans and mice (68). Aoi et al. examined gastrocnemius muscle from mice subjected to regular exercise and found that, compared with sedentary controls, SPARC secretion was significantly higher in the exercised group; moreover, regular low-intensity exercise effectively reduced colon cancer incidence (17). *In vitro* studies demonstrate that SPARC modulates tumor progression through multiple mechanisms, including suppression of cancer cell proliferation, induction of programmed cell death, and inhibition of cellular invasion and metastatic spread (20). However, all these conclusions were drawn from experiments using supraphysiological SPARC concentrations and did not account for the effects of physiological *in vivo* levels or the influence of the TME.

SPARC exerts antitumor effects through several distinct mechanisms. First, it interferes with growth factor signaling by reducing the binding efficiency of platelet-derived growth factor (PDGF) and transforming growth factor-β (TGF-β) to their respective receptors, thereby inhibiting DNA synthesis, arresting the cell cycle at G0/G1, and suppressing tumor cell proliferation (69). Second, SPARC inhibits EMT, which enhances the sensitivity of gastric cancer cells to radiotherapy and chemotherapy and improves therapeutic outcomes (70). Third, it induces apoptosis by activating the extrinsic apoptotic pathway: SPARC binds to the N-terminal domain of procaspase-8 to initiate the extrinsic cascade, which subsequently triggers the intrinsic pathway via Bid cleavage. This process ultimately leads to apoptosis in colon cancer cells and increases their chemosensitivity (71). Additionally, exogenous SPARC treatment significantly suppressed proliferation and promoted apoptosis in ovarian cancer cell lines such as SK-OV-3 (72). These experimental findings align with clinical observations showing that low SPARC expression correlates with higher tumor malignancy. Nevertheless, the precise signaling pathways and the upstream and downstream molecular networks mediating SPARC-induced apoptosis remain incompletely defined, and validation in clinical samples is still lacking.

In a pancreatic cancer model, overexpression of matrix metalloproteinase 9 (MMP-9) markedly enhanced cell migration and invasion, whereas exogenous SPARC treatment effectively counteracted this effect (73). Furthermore, SPARC inhibits tumor angiogenesis by downregulating vascular endothelial growth factor (VEGF) and matrix metalloproteinase 7 (MMP-7), thereby impairing the invasive and metastatic potential of gastric cancer cells (18). Although prior studies have established multiple anticancer roles for SPARC, the intrinsic connection between exercise-induced skeletal muscle secretion of SPARC and its function within the TME remains unclear. Moreover, the molecular underpinnings of the “exercise–muscle–SPARC–tumor” regulator*y* axis warrant further in-depth investigation.

#### 5.3.2 Irisin

Irisin is an exercise-responsive myokine secreted by skeletal muscle. Studies show that circulating irisin levels increase markedly after exercise, and irisin has demonstrated the potential to suppress malignant tumor phenotypes in various models by modulating multiple signaling pathways (74, 75). Huang et al. established a xenograft tumor model by subcutaneously inoculating glioblastoma U-87MG cells into the right abdominal region of thymus-deficient nude mice and found that exogenous irisin administration reduced tumor volume by more than 85% compared with the control group (26).

Irisin exerts tumor-suppressive effects through several key mechanisms. In U-87MG glioblastoma cells, irisin treatment significantly upregulates p21 mRNA and protein expression, resulting in cell cycle arrest at the G2/M phase (26). In pancreatic cancer models, irisin activates the AMPK signaling pathway in both MIA PaCa-2 and Panc03.27 cells by enhancing AMPK phosphorylation. This activation subsequently inhibits the downstream mTOR–p70S6K/4E-BP1 axis, contributing to its antitumor activity and leading to G0/G1 phase arrest and a marked reduction in cell proliferation (25). However, under identical treatment conditions, the degree of G0/G1 arrest varies substantially across pancreatic cancer cell lines, indicating that irisin's effects are cell line–specific and that the underlying mechanisms remain unclear.

In BxPC-3 pancreatic cancer cells, irisin treatment increases expression of the pro-apoptotic protein Bax while decreasing levels of the anti-apoptotic protein Bcl-2. This shift elevates the Bax/Bcl-2 ratio and promotes apoptosis (76). In breast cancer MDA-MB-231 cells, irisin enhances caspase-3/7 activity, confirming its role in activating the canonical apoptotic pathway (77).

EMT plays a pivotal role in tumor invasion and metastasis. In osteosarcoma models, irisin upregulates the epithelial marker E-cadherin by inhibiting STAT3 phosphorylation and disrupting the STAT3/Snail signaling axis. Concurrently, it downregulates mesenchymal markers, including N-cadherin and matrix metalloproteinases (MMP-2, MMP-7, MMP-9), thereby reducing cellular invasiveness (24). In lung cancer models, irisin suppresses EMT by modulating the PI3 K/AKT/Snail pathway and attenuates tumor cell migration (78). Additionally, irisin increases expression of tissue factor pathway inhibitor 2 (TFPI-2), a known angiogenesis inhibitor, thereby exerting anti-invasive and antimetastatic effects in glioblastoma (26).

Collectively, *in vitro* studies and animal models demonstrate that irisin exerts antitumor effects by regulating the cell cycle, inducing apoptosis, and inhibiting EMT. As an exercise-responsive myokine, it holds broad potential for cancer prevention and therapy. Nevertheless, the *in vivo* microenvironment is influenced by multiple factors, including immune regulation, metabolic status, and tissue specificity. Therefore, the stability, bioavailability, and long-term efficacy of irisin under physiological conditions require rigorous *in vivo* validation to support clinical translation.

#### 5.3.3 Core proteoglycan (decorin)

Decorin is a small leucine-rich proteoglycan that regulates collagen fiber assembly, cell proliferation, and adhesion. As a key myogenic factor, decorin also plays an important role in tumorigenesis. Studies report that decorin expression is low in various cancer cell lines, including those derived from colon cancer (13), breast cancer (15), and lung cancer (79). Elevating decorin levels effectively suppresses tumor cell growth and proliferation. In animal models, treatment with recombinant human decorin markedly reduces the growth, invasion, and metastasis of MTLn3 breast cancer cells in both nude mice and rats bearing orthotopic breast tumors (15).

Decorin exerts its antitumor effects primarily through three mechanisms. First, it antagonizes growth factor signaling pathways by binding to specific growth factors or cytokine receptors on the surface of tumor cells, such as the epidermal growth factor receptor (EGFR), thereby disrupting downstream signal transduction. This interference inhibits tumor proliferation, invasion, and metastasis while promoting apoptosis (80). For example, decorin binding to EGFR induces expression of the cyclin-dependent kinase inhibitor p21, leading to G1 cell-cycle arrest in A431 squamous cell carcinoma cells and consequent suppression of proliferation (81). Second, decorin suppresses EMT. In the colon cancer cell line LOVO, upregulation of decorin significantly reduces levels of EMT-associated transcription factors (Snail, Slug) and matrix metalloproteinases (MMP-2, MMP-9), enhances expression of the epithelial marker E-cadherin, and diminishes expression of the mesenchymal marker vimentin, thereby inhibiting invasion and metastasis (82, 83). Third, decorin impedes tumor angiogenesis by inducing autophagy in vascular endothelial cells. In non–small cell lung cancer A549 cells, this process disrupts vascular endothelial growth factor receptor 2 (VEGFR2) function, ultimately exerting antiangiogenic and antitumor effects (84).

In conclusion, decorin, a mechanoresponsive myokine, demonstrates significant antitumor potential across diverse cancer models, acting through modulation of key signaling pathways, inhibition of EMT, and suppression of tumor angiogenesis. Current evidence indicates that decorin predominantly exerts antitumor effects; however, the regulation of its *in vivo* secretion, the biological impact of physiological concentrations, and its tissue-specific modes of action remain to be fully elucidated.

## 6 Summary and outlook

**Figure 2:**
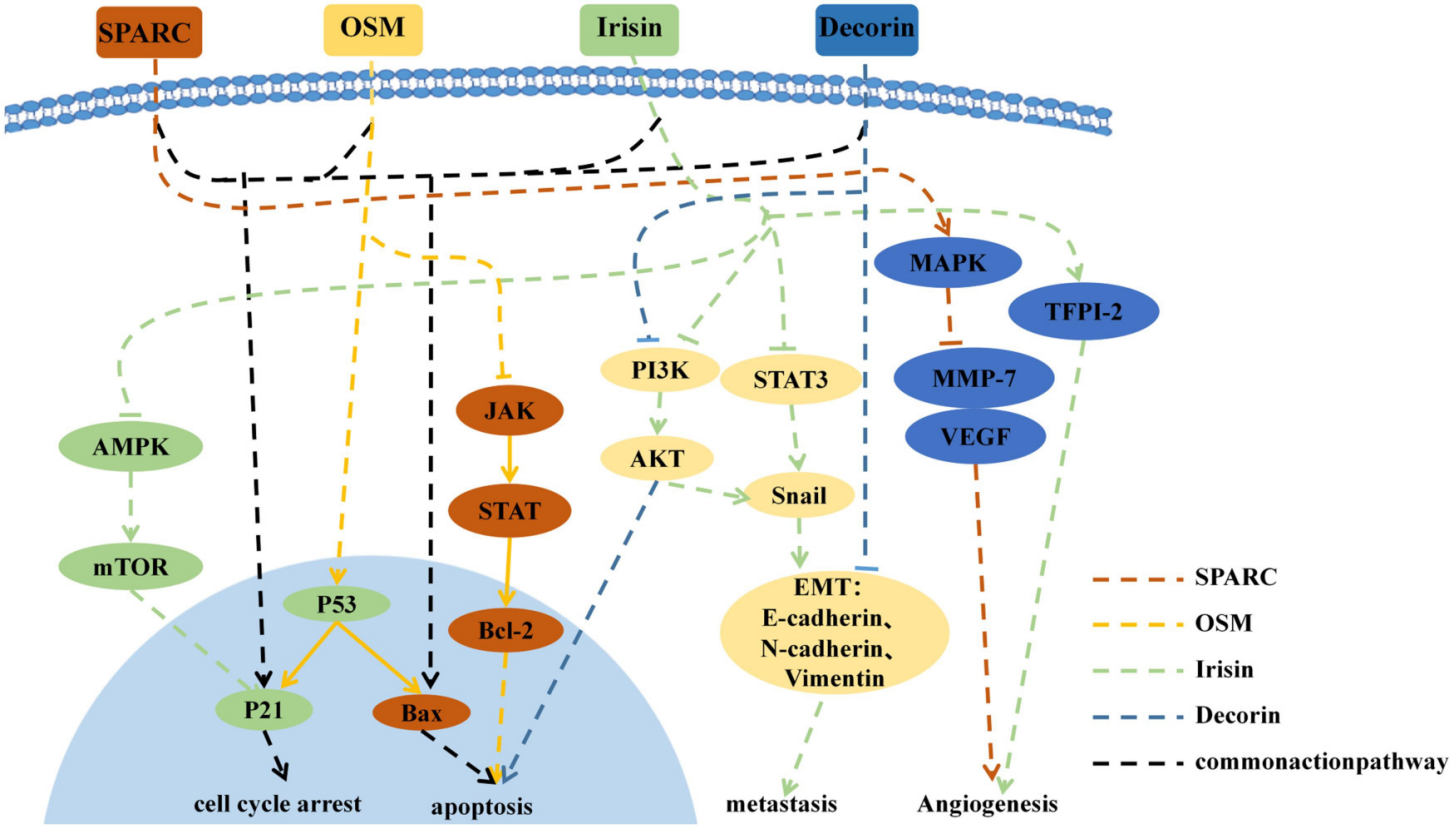
Schematic diagram of tumor inhibitory signaling pathway of myokines.

In conclusion, exercise serves as an effective nonpharmaceutical intervention and demonstrates significant value in cancer prevention and treatment. Skeletal muscle, the central component of the motor system, exerts antitumor effects through multiple pathways, with the secretion and activity of exercise-responsive myokines representing a key mechanistic link. This article systematically reviews the regulatory mechanisms governing the secretion of common exercise-responsive myokines, including cytokines, growth factors, and other proteins, and examines their molecular roles in inhibiting tumor cell proliferation, inducing apoptosis, suppressing invasion and metastasis, and modulating the TME. Acting through diverse signaling pathways and mechanisms, these myokines collectively constitute the essential biological basis for the anticancer effects of exercise. The interrelated regulatory networks are illustrated in Figure 2.

It is worth noting that most myokines exhibit considerable functional heterogeneity, with their effects strongly dependent on both the microenvironment and concentration. This remains a core issue that urgently requires critical evaluation in current research. Based on existing evidence, this paper classifies the myokines discussed into two categories according to their functional characteristics: (1) those primarily studied for their anticancer effects, and (2) those demonstrated to exert bidirectional regulatory effects in current studies. The specific classifications and functions are presented in Tables 2, 3.

**Table 2:** Myokines with anticancer effects studied in current research.

Myokine	Cancer models	Core mechanism (based on current evidence)
OSM	Breast cancer (16)	Activates STAT3/p53/p21 axis, induces cell cycle arrest, and inhibits proliferation
	Hepatocellular carcinoma (19)	
IL-7/IL-15	Breast cancer (57)	Promotes activation and proliferation of T cells and NK cells, enhancing antitumor immunity
IL-18	Colorectal cancer (58, 60)	Mediates pyroptosis-related immune activation and enhances NK cell cytotoxicity
SPARC	Colon cancer (17)	Inhibits proliferation, invasion, EMT and angiogenesis
	Prostate cancer (20)	
	Gastric cancer (70)	
	Colon cancer (71)	
	Ovarian cancer (72)	
	Breast cancer (85)	
	Gastric cancer (18)	
	Pancreatic cancer (18)	
Irisin	Glioblastoma (26)	Induces cell cycle arrest and apoptosis, inhibits EMT and metastasis
	Pancreatic cancer Panc03.27 (25)	
	Pancreatic cancer (76, 86)	
	Breast cancer (77)	
	Osteosarcoma (24)	
	Lung cancer (78, 87)	
Decorin	Liver cancer (90)	Antagonizes EGFR signaling, inhibits EMT and angiogenesis
	Breast cancer (15, 89)	
	Squamous cell carcinoma (82)	
	Colorectal cancer (82, 83)	
	Lung cancer (84)	
	Prostate cancer (88)	

**Table 3:** Myokines with bidirectional regulatory effects in current research.

Myokine	Cancer models	Core mechanism (based on current evidence)
IL-6	Breast cancer (27, 50)	Acute secretion induced by exercise suppresses tumors; chronic secretion in the TME promotes proliferation, invasion and immune escape
	Prostate cancer (51)	
	Melanoma (21)	
IGF-1	Prostate cancer (30, 62)	Most studies support a tumor-promoting role; exercise can regulate its secretion to suppress tumor cell growth
BDNF	Glioma (67)	TrkB pathway drives tumor progression; BDNF-AS and immune activation exert tumor-suppressive effects
	Head and neck squamous cell carcinoma (65, 66)	
IL-1β	Colorectal cancer (30, 61, 62)	Moderate expression enhances antitumor immunity; sustained high expression promotes inflammation and tumor progression

The current evidence base is primarily derived from *in vitro* cell experiments, which offer a relatively low level of evidence and limit direct extrapolation to human physiology. Most animal studies have employed immunodeficient models that fail to recapitulate a fully functional immune microenvironment. Clinical data remain extremely scarce, and large-scale, prospective, randomized controlled trials are lacking. Although research into the anticancer mechanisms of exercise-responsive myokines has advanced considerably, several scientific challenges persist, particularly regarding the regulation of myokine function and discrepancies between *in vitro* and *in vivo* findings, which warrant rigorous critical evaluation and further investigation.

First, the quantitative relationship between exercise parameters, such as type, intensity, duration, and frequency, and the resulting myokine secretion profile remains unclear, and the regulatory determinants of myokine functional heterogeneity have not been fully addressed. Certain myokines exhibit seemingly contradictory roles in tumor biology, simultaneously demonstrating both pro- and anti-cancer effects. This duality does not stem from an intrinsic binary nature of the molecules themselves but is largely shaped by their secretion concentration, temporal dynamics (e.g., acute pulsatile vs. chronic persistent release), and cellular origin (skeletal muscle–derived vs. TME–derived). However, current research has yet to delineate how different exercise parameters govern the functional directionality of myokines. There is an urgent need to develop a mathematical model that captures the dynamic interplay between exercise regimens and myokine secretion and function, grounded in standardized clinical cohort studies and rigorously controlled basic experiments. Such a model would provide a robust theoretical foundation for designing personalized exercise interventions while mitigating potential cancer-promoting risks.

Compounding this challenge, research into the synergistic regulatory network of myokines remains limited, and critical analyses of the mechanisms underlying their functional heterogeneity are lacking. Most studies have focused on individual muscle-derived factors and have not adequately explored inter-myokine signal exchange or cascade regulation. Moreover, the synergistic or antagonistic interactions among different myokines have rarely been examined in depth. For instance, some myokines display anti-cancer activity when administered alone, yet their combined action with other myokines, through concentration summation or signaling crosstalk, may shift the overall network toward a pro-tumorigenic state. This complex regulatory behavior has not been systematically investigated. Therefore, integrating multi-omics approaches (e.g., proteomics and transcriptomics) with systems biology methodologies is essential to uncover the global regulatory architecture and functional principles governing the myokine network.

Further complicating the picture, the role of EVs in mediating myokine transport remains poorly understood, and the impact of EV-mediated delivery on myokine function has received insufficient attention. The inherent biological properties of EVs, such as tissue targeting specificity and molecular stability, can significantly influence myokine delivery efficiency and may also modulate their functional outcomes. For example, EV-mediated targeted delivery of skeletal muscle–derived myokines could potentiate anti-cancer effects, whereas EVs carrying TME-derived myokines might exacerbate pro-tumorigenic signaling. This issue warrants further in-depth investigation to guide the clinical translation of myokine-based strategies and to minimize unintended cancer-promoting consequences.

Equally concerning is the heavy reliance of current research on *in vitro* cell systems, which necessitates more nuanced interpretation of experimental findings. This dependence partly accounts for inconsistent conclusions regarding myokine function. Most *in vitro* studies assess changes in the expression or activity of selected signaling molecules without fully reconstructing complete signal transduction pathways. Furthermore, these experiments often employ supraphysiological myokine concentrations in monoculture settings, failing to recapitulate key aspects of the *in vivo* microenvironment, such as immune homeostasis, oxygen tension, and stromal interactions. Consequently, tumor-suppressive effects observed *in vitro* may not reflect physiological responses *in vivo*. Myokines that demonstrate anti-cancer activity under artificial culture conditions may exhibit altered biological functions during prolonged *in vivo* exposure or within specific TME contexts. Additionally, *in vitro* models cannot replicate dynamic features like exercise-induced pulsatile myokine secretion and struggle to distinguish functional differences between myokines of distinct origins. These limitations impede a comprehensive understanding of the molecular mechanisms and regulatory logic underlying myokine-mediated anti-tumor effects.

Finally, existing *in vivo* research models suffer from significant constraints that hinder the clinical translation of myokines. The most commonly used animal models are immunodeficient mice, which cannot faithfully reproduce the intricate immune–tumor interaction networks present in human patients. Concurrently, the pharmacokinetic profiles of myokines, including their half-life, metabolic clearance pathways, and tissue distribution, remain inadequately characterized. Current *in vivo* delivery strategies for myokines also require substantial refinement. Unmodified (free) myokines exhibit poor stability and limited targeting capacity, often failing to achieve therapeutically effective concentrations at tumor sites. Potential off-target effects on healthy tissues remain insufficiently assessed, and dedicated safety studies are still scarce. Clinical evidence is equally limited: large-scale, prospective trials validating the efficacy and safety of myokine-based interventions are lacking, thereby constraining their path toward clinical application.

Advancing functional studies *in vivo* and validating key findings in clinical samples represent critical priorities for the future development of this field and a pivotal step toward resolving ongoing controversies surrounding myokine functionality. *In vivo* models that more faithfully recapitulate human physiology are essential to elucidate the mechanistic roles of myokines within complex organismal contexts, clarify how their functional outcomes vary with cellular source, concentration, and duration of exposure, and ultimately define the precise conditions under which they exert anti-tumor effects. Moving forward, efforts should prioritize the development of immunocompetent animal models, systematic pharmacokinetic characterization, optimization of targeted delivery platforms, and rigorous assessment of off-target effects, collectively generating the foundational data required for clinical translation. Key translational questions, including effective therapeutic dose ranges, metabolic stability, potential adverse reactions, and interindividual variability in human responses to myokines, must be addressed through well-designed, high-quality clinical studies. Concurrently, a comprehensive and unbiased synthesis of existing evidence is urgently needed to mitigate interpretive biases stemming from overreliance on *in vitro* data and to establish a more robust scientific basis for the clinical application of myokine-based strategies.

As molecular biology, immunomics, and precision medicine continue to converge, researchers are poised to achieve a more integrated understanding of the molecular mechanisms underlying exercise-induced myokine-mediated anti-cancer effects and to delineate the regulatory principles governing myokine functional plasticity. Such advances will not only provide a theoretical foundation for developing kinesiology-inspired anti-tumor therapeutics and designing personalized exercise interventions but also foster deeper interdisciplinary collaboration between sports medicine and oncology. By critically evaluating current evidence and proactively mitigating potential cancer-promoting risks associated with myokine signaling, the field stands to make transformative contributions to cancer prevention and therapy, ultimately improving outcomes for a broad spectrum of patients.
